# Assessing effectiveness of exclusion fences in protecting threatened plants

**DOI:** 10.1038/s41598-021-95739-4

**Published:** 2021-08-09

**Authors:** Juan Lorite, Carlos Salazar-Mendías, Roza Pawlak, Eva María Cañadas

**Affiliations:** 1 Department of Botany, University of Granada, 18071 Granada, Spain; 2 Interuniversity Institute for Earth System Research, University of Granada, 18006 Granada, Spain; 3 Department of Animal Biology, Plant Biology and Ecology, University of Jaén, 23071 Jaén, Spain

**Keywords:** Conservation biology, Environmental impact, Plant ecology

## Abstract

Overgrazing stands out as threat factors on biodiversity, being especially harmful in the Mediterranean, due to strong human pressure and an accelerated climate change acting synergistically. Fencing is a common tool used in conservation biology to tackle this problem. Advantages of fences are usually fast, intuitive, and easy to evaluate. However, disadvantages could also arise (increasing interspecific competition, disturbing habitat structure, limiting pollination, reducing dispersion). Together with management issues (maintenance, conflicts with stakeholders, and pulling effect). Effectiveness of fencing for conservation has been frequently assessed for animals, while it is almost a neglected topic in plants. We evaluated the outcome of fencing three threatened and narrow-endemic plants. Selected 5 populations were only partly fenced, which allowed comparing different variables inside and outside the fence. For evaluating the fencing effects, we sampled several habitats (vegetation cover, composition, density of target species), and target-species features (individual size, neighbouring species, and fruit-set). Fencing had strong effects on the habitat and on target-species individuals, showing contrasting responses at species and population level. Particularly, for *Erodium cazorlanum*, fence had a positive effect in one case, and negative in another. In *Hormathophylla baetica* effect was positive in all populations. Finally, fencing negatively affected *Solenanthus reverchonii* by increasing competition and limiting seed-dispersal. Fencing outcome was different in assessed species, highlighting the need to a case-by-case evaluation to determine the net balance (pros vs. cons), also its suitability and most favourable option (i.e. permanent vs. temporary fences).

## Introduction

Land-use activities have transformed a large proportion of the planet’s land surface becoming an important driver of the ongoing global change^1^. Within the land-use activities overgrazing, both wild- and domestic-animals caused, is among the most detrimental ones. In fact, it has strong links with erosion, biodiversity loss and desertification in large areas worldwide^1,2^. Nowadays, overgrazing is an important threatening factor for plant species, especially for endemic ones due to their restricted distribution area, usually encompasses with low population sizes^3^. Despite this general pattern, some endemic and/or threatened species could be favoured by a high grazing pressure, or even overgrazing that limit interspecific competition^4^, or creating a window of opportunity for some species (i.e. annual species)^5^.

One of the most used tools since the Neolithic Age to protect plants (usually crops) from the herbivores is fencing^6^. In conservation biology fencing is one tool of the conservation managers’ arsenal that can separate threats from biodiversity. Fencing is referred not only to physical barriers, such as a standard post and wire fence, but also metaphorical ones, such as inhospitable land, gardens of chilli, walls of noise, effective anti-poaching patrols or buffers of poison^7^. The idea is so simple: the separation of biodiversity (plants in this case) from the processes threatening them (herbivory)^8^.

Usually, positive outcomes of fencing plants are very fast, easy to evaluate and hence very intuitive. Fencing has a clear positive effect by avoiding herbivore grazing and trampling, also in some cases for human trampling and collection^9,10^. This usually results in an increasing of seed production and seedling survival at short term^10–12^. However, at medium or long term fencing produces important changes in vegetation composition and structure^4^. These drastic changes may have negative effects on a species such as: increasing intraspecific competition, limitation of seed dispersal for some zoochorous species^13^, and producing changes in population gene flow^14^. Also, it has indirect effects such as changes in pollination fauna and pollen dispersal due to these drastic changes in the habitat^14^. Hence, many times the net outcome is difficult to foresee and even difficult to address. Moreover, fences produce aesthetic problems and management problems with stakeholders (e.g. farmers or hunters), or call effect for illegal collectors^8^. Altogether with high costs associated not only with installation, but also to their maintenance, that could not be affordable in most of extensive conservation programs^15,16^.

Although the pros and cons of fencing for conservation have largely assessed for animals, especially for large mammals (see^8^, for a review), the effects in plant conservation/protection against the herbivores are more scarce in literature.

Mediterranean ecosystems represent paradigmatic examples of this problem. They have been used for farmers since Neolithic and they constitute a hotspot of biodiversity with a large number of endemic species, many of them threatened by different factors mainly with anthropogenic origin. Among them, one of the most important threat factors is overgrazing^17,18^.

In this context, Sierra de Cazorla is an outstanding area within the Baetic-Rifan hotspot^19^. In this area there is a large number of herbivores both wild and domestic that produce overgrazing problems in some areas, especially in dry years. For this reason, since the middle 1980s some populations of threatened species were fenced^20^.

The results of pre-existing studies as well as our observations in this and other territories do not provide clear evidence regarding the suitability of this tool for managing threatened populations. Thus, it is crucial to assess these fenced populations in order to extract lessons for planning the installation of new fences, prioritizing the maintenance of the existing ones, or even the removal of the most detrimental for some endangered species.

We hypothesize that the effects of plant fencing as a tool to stop the threat of herbivory depends on both the site and the species. Thus, we aimed to assess fencing-induced changes at the individual, microhabitat and habitat levels in several populations of three threatened plant species.

## Results

Unfenced areas presented an overall mean vegetation cover of 46.82 ± 3.31% (ranging from 20 to 80%), while for fenced areas cover was 54.69 ± 3.06% (28–88%). PERMANOVA showed significant differences in species composition based mainly in populations (df = 4; pseudo F = 28.84; P < 0.001), but also in species (df = 2; pseudo F = 19.18; P < 0.001), and fencing/no fencing (df = 1; pseudo F = 3.85; P < 0.005).

**Figure 1:**
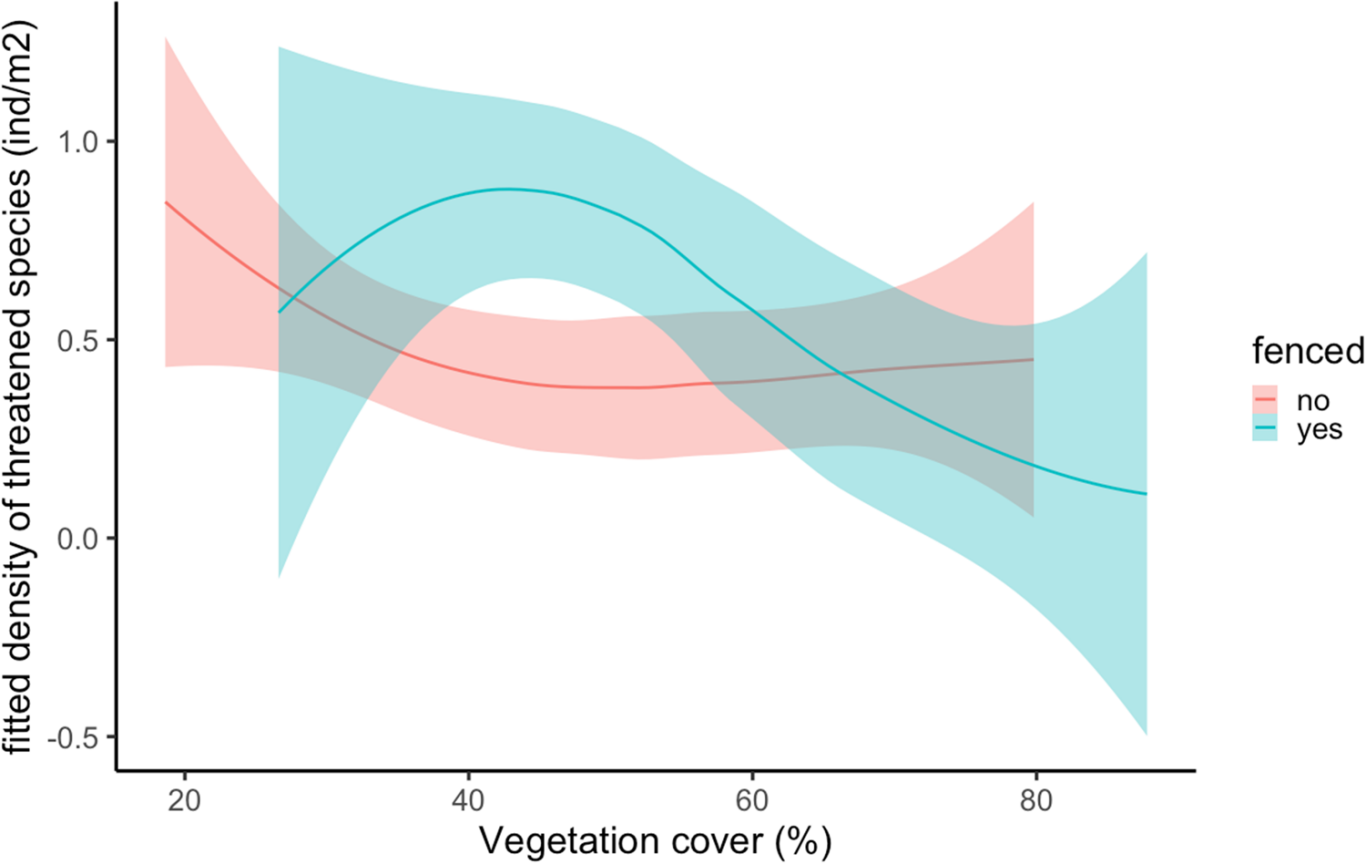
Relationship between vegetation cover (%) and density (individual/m^2^) of the focal threatened species for fenced (blue) and unfenced (red) habitat patches (fitted values adjusted by linear mixed model for all the species and populations, see Table S2).

Relationship between focal species density and vegetation cover, regardless population and species, showed significant differences for fenced/unfenced populations (Tables S2, S3). At unfenced patches focal individuals tended to be more dense at lower cover values and remained with the same density along the cover gradient (Fig. 1). Meanwhile at fenced ones, intermediate values of vegetation cover presented a higher density for focal individuals, whereas at higher cover values (> 60%) density dropped sharply for fenced patches.

**Figure 2:**
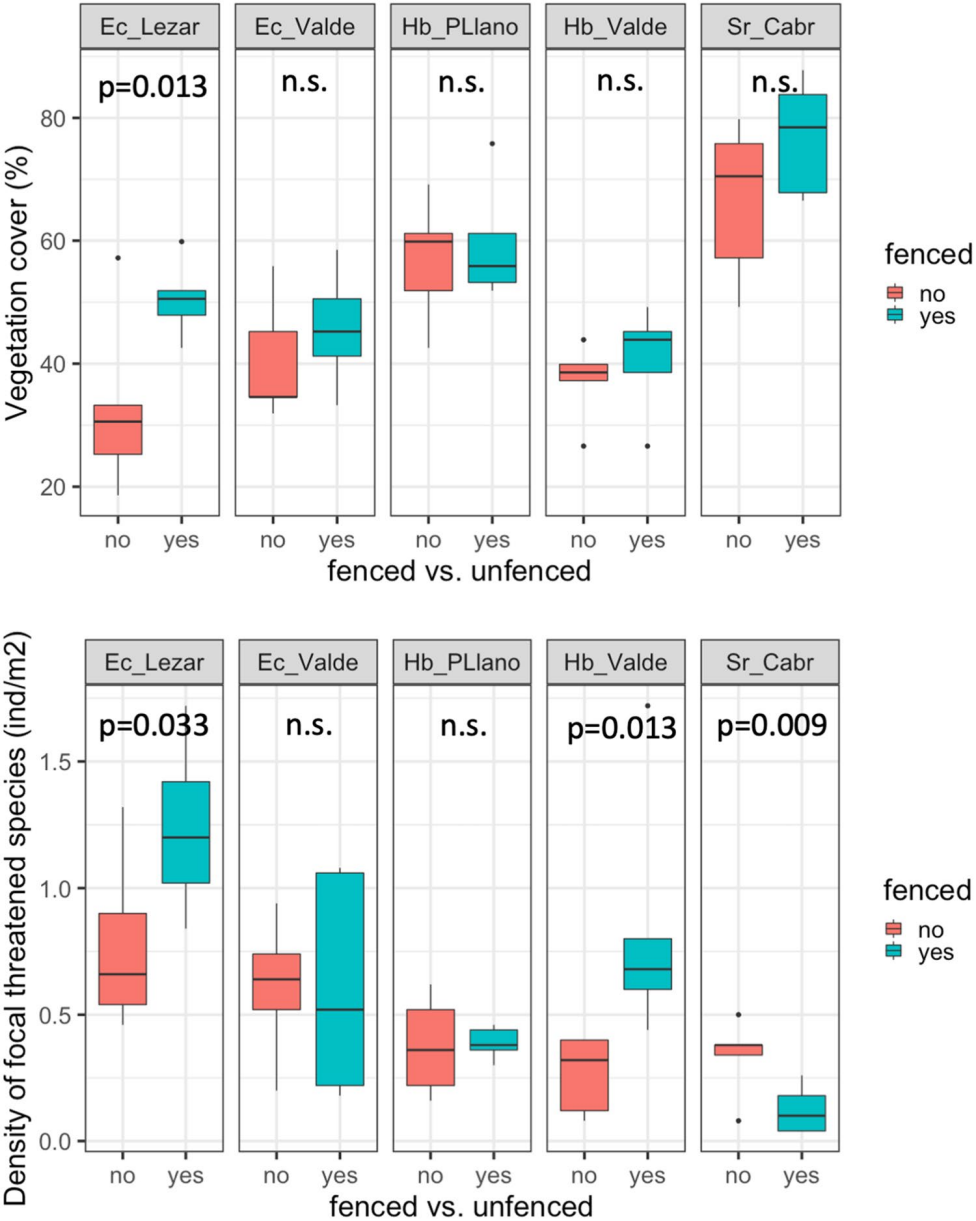
Boxplot for cover (top graphs) and density (bottom graphs) of fenced and unfenced habitat patches for all the studied populations (see Table S1). P values indicated for each pair comparison were obtained after permutational ANOVA. Abbreviations for species and populations: *Ec_Lezar* *Erodium cazorlanum*-Puerto de Lezar, *Ec_Valde* *Erodium cazorlanum*-Valdeazores, *Hb_PLlano* *Hormathophylla baetica*-Puerto Llano, *Hb_Valde* *Hormathophylla baetica*-Valdeazores, *Sr_Cabr* *Solenanthus reverchonii*-Cabrilla Alta.

All the populations showed similar vegetative/reproductive ratios for fenced and unfenced patches (Figure S2), except for *Solenanthus reverchonii* where around 13% of fenced individuals flowered and fructified, while unfenced ones behaved as vegetative in all cases. For the analysed traits, we obtained significant differences for fenced/unfenced populations in some cases, both among species and among populations within species. For vegetation cover (Fig. 2) we did not obtain significant differences between fenced and unfenced populations, except for one *E. cazorlanum* population (Puerto de Lezar; P = 0.013). Also, individual density of focal species did not show a consistent pattern across populations (Fig. 2), while some populations presented a higher density in fenced patches other populations did not show significant differences. For *Solenanthus reverchonii* fenced population showed a lower density in comparison with unfenced areas (P = 0.009).

**Figure 3:**
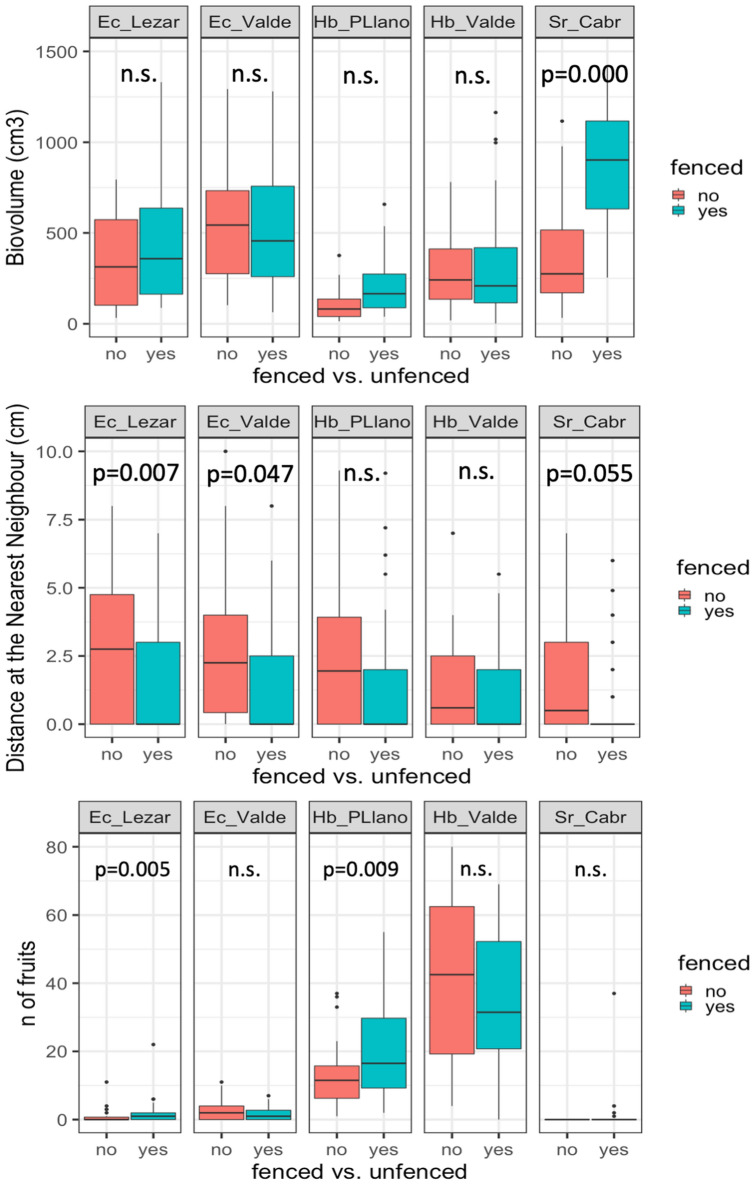
Boxplot of biovolume (top graphs), distance to the nearest neighbour (centre graphs), and number fruit production (bottom graphs) for fenced and unfenced individuals (n = 30 in all the cases) in all the studied populations (see Table S1). P values indicated for each pair comparison were obtained after permutational ANOVA. Abbreviations for species and populations: *Ec_Lezar* *Erodium cazorlanum*-Puerto de Lezar, *Ec_Valde* *Erodium cazorlanum*-Valdeazores, *Hb_PLlano* *Hormathophylla baetica*-Puerto Llano, *Hb_Valde* *Hormathophylla baetica*-Valdeazores, *Sr_Cabr* *Solenanthus reverchonii*-Cabrilla Alta.

When analysing biovolume as surrogate of plant biomass (Fig. 3) we did not obtain significant differences in fenced and unfenced patches, except for *Solenanthus reverchonii*, where fenced plants tend to be significantly bigger (P = 0.000). In fact, no regeneration was found inside the fence and plants tend to be more scarce but bigger in size. Distance to the nearest neighbour (Fig. 3) increased in two populations, while sharply decreased in *Solenanthus reverchonii* (P = 0.055).

Lastly, fruit production (Fig. 3) showed differences in just two populations (*E. cazorlanum*-Lezar, P = 0.005 and *H. baetica*-Valdeazores, P = 0.009). Remarkably, in both cases unfenced populations produced significantly more fruits than fenced ones.

## Discussion

As above stated, fencing is a commonly used tool in conservation biology^8,21^. It is assumed that fencing has a positive effect over biodiversity, given that short-term response in plants is usually positive by avoiding herbivore grazing and trampling, human trampling, or collection^9,11^. Even though, this assumption is usually poorly supported by empirical data^22^. In fact to evaluate these long-term trends, even at short-term, is usually a neglected issue^21^. In our study, for the three species analysed fencing has not always had positive consequences over the evaluated response variables at mid-term. In fact, we obtained contrasting results not only for species, but also for populations within a given species. Hence, medium or long-term effects of fences usually are related with complex both population and community dynamics^23^, being not so straightforward, and supporting the idea that net results in mid or long term is species- and habitat-dependent^8^. In some cases, moderate intensity trampling and grazing proved to be useful at medium term to limit the interspecific competition and undesirable changes in the vegetation structure for the target species^10^. Also, effect of grazing and trampling could greatly differ among species and populations within species, switching from positive to negative with complex interactions between them^11,24^.

One of the most influential factors in the net outcome of fencing is the change in vegetation cover in absence of herbivory, that may lead important changes in community structure and function^23^. In our study, the best performance for fenced populations was obtained at intermediate vegetation cover values, while for patches that become more densely vegetated after fencing, target species populations tend to significantly decrease, in line with other research results^21^. Herbivory alter vegetation cover and structure^25^. Hence, in absence of herbivory interspecific competition tend to increase significantly^25,26^, which may cause the negative consequences sometimes observed at mid or long term, especially for undercanopy species^21^, conducting to a homogenization of plant community^23^.

In general, altitude (as an arrange of different associated abiotic and biotic factors,^27^) is very influential in controlling an excessive increase in vegetation cover. In our cases, fenced high-mountain populations tended to perform better than in middle areas, as in high mountain areas environmental constrains limit cover and consequently the interspecific competence^28^. As a rule of thumb, we could establish that fences will exert better results for target species on naturally open habitats.

Habitat or microhabitat disparity in a given species may provoke differences in fencing performance^13^. For our target species, indicator variables (i.e. cover, density of focal species, biovolume, distance to the nearest neighbour, and fruit production) showed contrasting results for the populations within the same species and for the different indicator variables within the same population. Moreover, in most of the populations net outcome of the different features was not significantly different for fenced and unfenced species. This lack of a clear positive effect at mid-term over studied species do not justify the high costs associated with fencing installation and maintenance^15,16^. Together with potential conflict with stakeholders that may entail fencing^8^.

In some cases fencing could be even particularly detrimental for species^14^. We found a quite detrimental effect for *Solenanthus reverchonii*, with a declining tendency in fenced patch. This fact relies on the very low fruit production, no recruitment and an increased interspecific competence (high vegetation cover and virtually no distance with the nearest neighbour). Interestingly, this species is exozoochorous (i.e. Dispersal of seeds by being carried on the surface of an animal)^18^, thus the drastic change in habitat structure, sometimes becoming virtually impenetrable, may have restricted or even arrested seed dispersal^13^. Afterwards, seeds could not find suitable open sites typically inhabited by the species^18,20^. In this case fencing seemed to be positive at short term^20^, but over the years the population trend has become clearly negative.

The present study is focused in three species with different biotypes and contrasted habitats. However, the low number of species do not allow us to generalize our results. Also, to have detailed and quantitative information about the grazing pressure, would be very helpful. This lack of information for the area is clearly a limitation of the present work. Another important issue is the complete absence of the data at the starting point. Differences appearing prior to the fence installation may be influential even after 17–35 years. In this sense, to compile detailed information about the habitat and target species at the starting point is crucial to evaluate the mid-term and long-term effects. Also, the presence of the fence could favour seed production and act as seed source dispersed to unfenced adjacent areas (i.e. for anemochorous or entomochorous seeds), thus minimizing the differences between fenced and unfenced areas. However, no apparent edge effect has been detected for habitat or target species variables. Despite these limitations, our study reveals that a previous evaluation (using the best available information), and a continuous monitoring is essential, not only at the short-term but also at mid- and long-term^23^. Also, for some species transformation of permanent fences in temporary ones could help to overcome partly the negative long-term effects of the total herbivory exclusion over community and population structure of the threatened species.

We have to rethink and re-evaluate all the existing fences, seeking for both ecological and economic viability. Furthermore, for establishing new fences an evaluation system must be implemented. In this regard, a plant functional type approach can be adopted^29^, compiling all the existing experiences, the scant literature, grey literature, and technical reports. Also, bearing in mind that fencing is only an emergency solution, and comprehensive measures, such as herbivore control (both wild and domestic) are always preferable despite controversial. Ultimately, grazing problem is just one of the consequences of the termed *tragedy of the commons*^30^.

## Methods

### Study area

The Sierra de Cazorla mountains s.l. are located in the northern part of the Baetic Mountain System (southeastern Spain, 38° 05′ N/2° 45′ W). Namely Cazorla, Segura, and Las Villas Natural Park, constitutes the largest protected area in Spain, covering 209,921 ha. The climatic regime is typically Mediterranean characterized by a hard summer drought. Average rainfall is about 1100 mm/year (ranging from 400 to 1900 mm), November and April being the wettest months, and July and August the driest ones, with marked interannual differences. Average temperature is 11.7 °C, with minimum in January (4 °C) and maximum in August (21 °C). Lithology consists mainly of limestone and dolomite^31^. A craggy topography characterizes these mountains, with altitudes ranging from 500 to 2107 m a.s.l. (*Empanadas* peak). The vegetation is composed of a mixture of pine forests (*Pinus halepensis* Mill.*, P. pinaster* Ait. and *P. nigra* subsp. *salzmannii* (Dunal) Franco) with broad lived perennial or deciduous oaks such as: *Quercus ilex* L. *and Q. faginea* Lam.^32^. The total amount of the vascular plants accounted for the area are 2200, with 360 endemics to the Baetic-Rifan complex, 35 among them being narrow endemics to these mountains^33^. This mountain range has been overgrazed by domestic and wild ungulates at least during the last century^34–36^, affecting significantly the structure, composition and regeneration of the vegetation^37^. In fact, this is the main threat factor for endangered plant species in the area^38^.

### Studied species

We selected three endemic and threatened species (nomenclature as in^39^) *Erodium cazorlanum* Heywood, *Hormathophylla baetica* Küpfer, and *Solenanthus reverchonii* Degen. Species belong to important families in the area and their biotypes (i.e. Hemicriptophytes and chamaephytes) are dominant for threatened species (see Table 1). Also, all of them are threatened mainly by overgrazing and their populations have been partially fenced for 15–37 years (Table S1) with the so-called sheep fences or hog fences (see Figure S1) intended to avoid grazing by ungulates that mainly damage the populations of target species (see references above). This allows us to compare several habitat, microhabitat and target-species features (both habitat structure and reproductive) for fenced and unfenced patches blocking the rest of uncontrolled environmental variables. Using the information in the Regional Database for threatened flora^40^, we selected all the existing partially fenced populations with well-maintained fences, and having all of them about 50% of the individuals outside the fence., i.e. two populations for *E. cazorlanum* and *H. baetica*, and one for *S. reverchonii* (Table S1). Moreover, in absence of data at the starting point for crucial variables such for the community (i.e. grazing pressure, community composition) or individuals of target species (see variables below), this design allow us to compare between fenced and unfenced individual within the same population. The habitat for all the selected populations was an open pine woodland (*Pinus nigra* subsp. *salzmannii*) with short shrubs (frequently cushion-like shape shrubs^41^).

**Table 1:** Main features of the studied species^18,20,38,44^.

	*Erodium cazorlanum*	*Hormathophylla baetica*	*Solenanthus reverchonii*
Family	*Geraniaceae*	*Brassicaceae*	*Boraginaceae*
Biotype	Hemicryptophyte	Chamaephyte	Hemicryptophyte
Flowering	Apr–May/Jul–Sep	Apr–May	Apr–May
Pollination type	Biotic (insect)	Biotic (insect)	Biotic (insect)
Fruiting	Jun/Sep–Oct	Jun	Jun–Jul
Seed dispersal mode	Wind-dispersed and autochorous	Passive (barochorous)	Biotic (exozoochorous)
Clonal reproduction	No	No	Stolons
Distribution range	Narrow endemic (Cazorla mountains)	Narrow endemic (Cazorla mountains)	Narrow endemic (Cazorla mountains)
Altitudinal range	1500–2100 m. asl	1500–2000	1700–1900
Populations	15	19	2
n of individuals	15,000 approx	3500 approx	340
Main threats	Overgrazing (grazing and trampling) Natural (restricted habitat)	Overgrazing (grazing and trampling) Natural (restricted habitat)	Overgrazing (grazing and trampling) Natural (restricted habitat)
Threat category	Vulnerable (VU)	Vulnerable (VU)	Critically endangered (CR)

### Sampling design

We performed the sampling from May to September 2016 (*H. baetica* and *S. reverchonii*) and May 2017 (for *E. cazorlanum*).

First, to evaluate the effect of fencing over the habitat we placed 10 transect of 25 × 2 m (50 m^2^), 5 inside the fence and 5 outside the fence per species and population (50 transects in total). To assess the cover per species we used the intercept point method^42^ with 3-point contacts per meter (one in the centre and two in each side 1 m apart), 75 points per transect in total, so the cover per species was estimated as the percentage of point occupied by a given species. Also, density (individuals per square meter) of focal species (i.e. the three threatened species) was calculated by counting all the individuals in each 25 × 2 m (50 m^2^) band.

To assess the changes in the microhabitat of the threatened species (i.e. vicinity of each individual of the threatened species) we randomly selected 30 individuals inside the fence and 30 outside per population and species (60 individuals per populations × 5 populations = 300 individuals in total). For each individual, we placed a circular plot of 50 cm in diameter with focal individual in the central point. In each circular plot we recorded the frequency of all the perennial species, distance, and the identity of the nearest neighbour. Also, we recorded the fruit set of the focal individual, as well as its height and average diameter in order to calculate biovolume as the semispheroid formed^43^.

For identification and naming of the encountered species we followed Vascular Flora of Eastern Andalusia^44^.

### Data analysis

Statistical analyses were performed using R version 3.6.1^45^.

We explored changes in species composition among populations both fenced and unfenced by means of multivariate analyses. First, we explored the influence of the categorical factors (i.e. species, population and fencing) over the matrix of species cover per transect by means of a permutational multivariate analysis of variance (PERMANOVA, Euclidean distance, with 999 permutations), by means of “adonis” function in R package vegan 2.5–2^46^. Relationship among cover and density for fenced vs unfenced patches for all the target species pooled was modelled by means of linear mixed models using “nlme” package^47^, including population and fence (fenced vs. unfenced) as fixed factors and transects nested in population as random factor. Model suitability was assessed by graphical exploration of the residuals^48^. To address differences in vegetation cover, density of each focal species, distance to the nearest neighbour, and fruit production, within each population for fenced and unfenced patches, we performed permutational ANOVAs by means of “lmPerm” package^49^, a flexible and very robust analysis that could cope with heteroscedasticity and a wide variety of statistical distributions. For graphs included we used ggplot2 package^50^. Throughout the text means are expressed ± 1 SE.

### Ethical approval

Authors declare that manuscript complies with all the institutional, national, and international guidelines and legislation, regarding all species and habitats studied.

## Supplementary Information

Supplementary Information 1.
