# Supplementary material for: Assessing effectiveness of exclusion fences in protecting threatened plants
Source: Sci Rep. 2021 Aug 9;11:16124. doi: 10.1038/s41598-021-95739-4 (PMC8352956; doi:10.1038/s41598-021-95739-4)
Supplement: Supplementary file 1 — Supplementary Information 1. [file 41598_2021_95739_MOESM1_ESM.docx]

Table S1. Details of the selected populations including fenced and unfenced patches.

| **Species** | **Population** | **Code** | **Altitude (m.a.s.l.)** | **Fencing**  **year** | **Total N ind.** | **N fenced ind.** |
| --- | --- | --- | --- | --- | --- | --- |
| ***Erodium cazorlanum*** | Puerto de Lezar | Ec_Lezar | 1898 | 1999 | 200 | 100 |
|  | Valdeazores | Ec_Valde | 1560 | 2004 | 661 | 302 |
| ***Hormathophylla baetica*** | Puerto Llano | Hb_Pllano | 1809 | 2005 | 360 | 234 |
|  | Valdeazores | Hb_Valde | 1560 | 2004 | 887 | 400 |
| ***Solenanthus reverchonii*** | Cabrilla Alta | Sr_Cabr | 1770 | 1983 | 240 | 62 |

Table S2. Summary of the linear mixed model for density of focal species (ind/m^2^). Significant differences at p<0.05 in bold. (Model= density ~ population*fenced, random= ~transect/population). Abbreviations for species and populations: Ec_Lezar=*Erodium cazorlanum*-Puerto de Lezar, Ec_Valde=*Erodium cazorlanum*-Valdeazores, Hb_PLlano=*Hormathophylla baetica*-Puerto Llano, Hb_Valde=*Hormathophylla baetica*-Valdeazores, and Sr_Cabr= *Solenanthus reverchonii*-Cabrilla Alta.

|  | **Response=density** | | |
| --- | --- | --- | --- |
| *Predictors* | *Estimates* | *CI* | *p* |
| (Intercept) | 0.78 | 0.51 – 1.04 | **<0.001** |
| population [Ec_Valde] | -0.17 | -0.54 – 0.21 | 0.370 |
| population [Hb_PLlano] | -0.40 | -0.77 – -0.03 | **0.037** |
| population [Hb_Valde] | -0.51 | -0.89 – -0.14 | **0.009** |
| population [Sr_Cabr] | -0.44 | -0.81 – -0.07 | **0.023** |
| fenced [yes] | 0.46 | 0.09 – 0.84 | **0.017** |
| population [Ec_Valde] *fenced [yes] | -0.46 | -0.99 – 0.07 | 0.087 |
| population [Hb_PLlano] * fenced [yes] | -0.45 | -0.98 – 0.08 | 0.093 |
| population [Hb_Valde] * fenced [yes] | 0.12 | -0.41 – 0.65 | 0.650 |
| population [Sr_Cabr] * fenced [yes] | -0.68 | -1.21 – -0.15 | **0.014** |
| N _tr_ | 50 | | |
| Observations | 50 | | |

Model AIC= 55.45826.

Model Rsquare= 0.5896519

Random effects: StdDev Intercept: 0.2745345; StdDev Residual: 0.1029504

Table S3. Summary of the ANOVA performed after the lineal mixed model for density (table S2) of focal species (ind/m^2^). Significant differences at p<0.05 in bold. (Model= density ~ population*fenced, random= ~transect/population).

| **Source** | **numDF** | **denDF** | **F-value** | **p-value** |
| --- | --- | --- | --- | --- |
| (Intercept) | 1 | 40.00 | 180.57 | **0.00** |
| population | 4 | 40.00 | 10.00 | **0.00** |
| fenced | 1 | 40.00 | 4.22 | 0.05 |
| population:fenced | 4 | 40.00 | 3.32 | **0.02** |

Figure S1. Partial view of sheep fence installed to protect *Erodium cazorlanum* at Valdeazores population (see table S1).


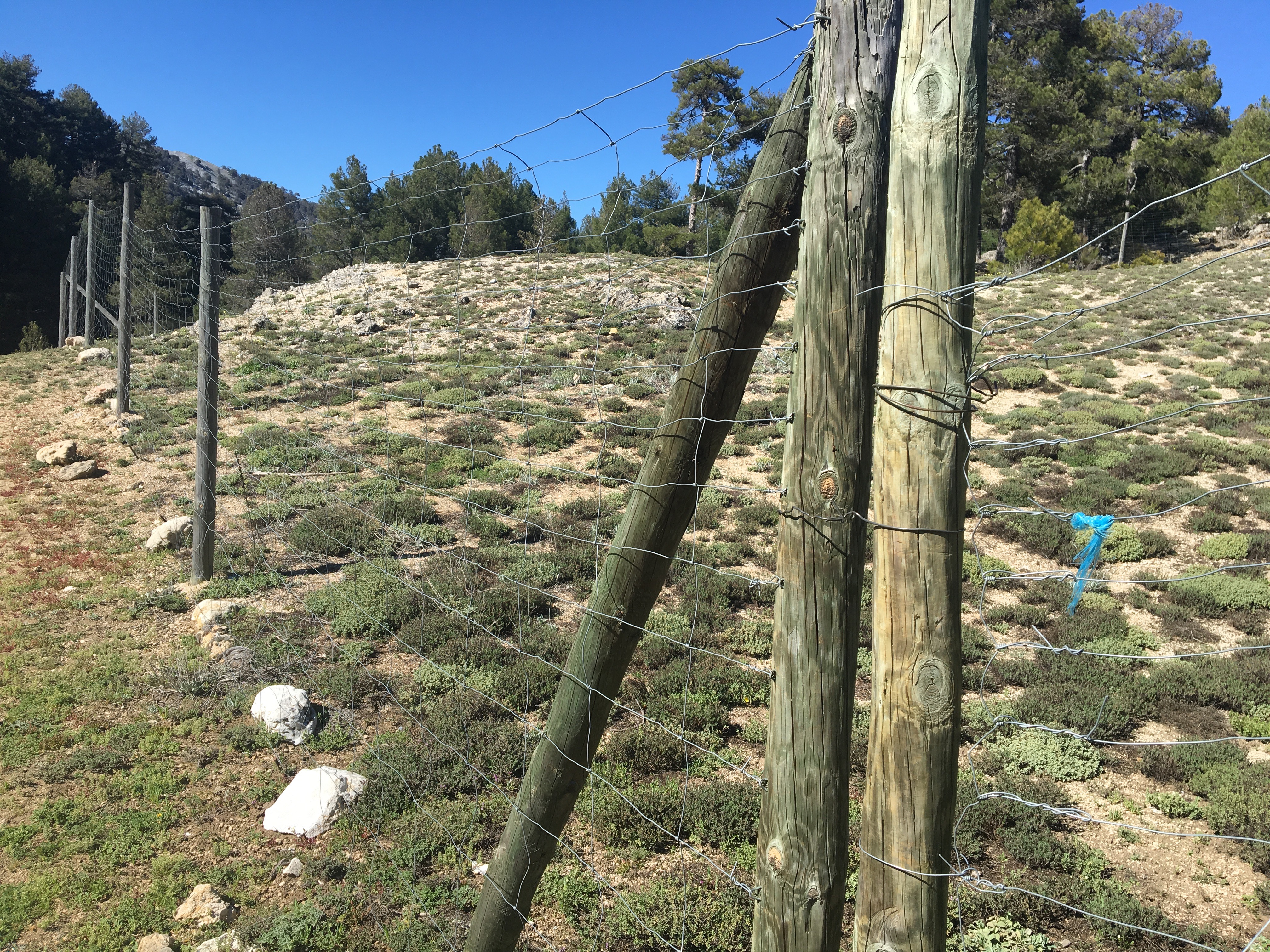


Figure S2. Relative percentage of vegetative (veg.) vs. reproductive (rep) for fenced (yes) and unfenced (no) individuals of the target species per population (Ec_Lezar=*Erodium cazorlanum*-Puerto de Lezar, Ec_Valde=*Erodium cazorlanum*-Valdeazores, Hb_PLlano=*Hormathophylla baetica*-Puerto Llano, Hb_Valde=*Hormathophylla baetica*-Valdeazores, and Sr_Cabr= *Solenanthus reverchonii*-Cabrilla Alta). n=30 in all cases.
